# Predictors of postoperative complication and prolonged intensive care unit stay after complete pericardiectomy in tuberculous constrictive pericarditis

**DOI:** 10.1186/s13019-020-01198-9

**Published:** 2020-06-19

**Authors:** Likui Fang, Guocan Yu, Jinpeng Huang, Wuchen Zhao, Bo Ye

**Affiliations:** 1 Department of Thoracic Surgery, Hangzhou Red Cross Hospital, Hangzhou, 310003 China; 2 Department of Nursing, Hangzhou Red Cross Hospital, Hangzhou, 310003 China

**Keywords:** Tuberculous constrictive pericarditis, Complete pericardiectomy, Postoperative complication, Intensive care unit stay

## Abstract

**Background:**

The risk factors of postoperative outcomes after pericardiectomy in tuberculous constrictive pericarditis have still been unclear. This study aimed to investigate the predictors of postoperative complication and prolonged intensive care unit (ICU) stay in the patients with tuberculous constrictive pericarditis undergoing pericardiectomy.

**Methods:**

A total of 88 patients with tuberculous constrictive pericarditis undergoing pericardiectomy were retrospectively enrolled. Logistic regression and Cox regression analysis were performed to identify the predictors of postoperative complication and prolonged ICU stay, respectively.

**Results:**

All patients underwent complete pericardiectomy and 35 (39.8%) had postoperative complication with no mortality within 30 days after surgery and no in-hospital deaths. Postoperative complication prolonged postoperative ICU stay (*P* < 0.001), duration of chest drainage (*P* < 0.001) and postoperative hospital stay (*P* < 0.001). Preoperative NYHA functional class (*P* = 0.004, OR 4.051, 95%CI 1.558–10.533) and preoperative central venous pressure (CVP) (*P* = 0.031, OR 1.151, 95%CI 1.013–1.309) were independent risk factors of postoperative complication. Postoperative complication (*P* < 0.001, HR 4.132, 95%CI 2.217–7.692) was the independent risk factor for prolonged ICU stay.

**Conclusion:**

Complete pericardiectomy was associated with high risk of postoperative complication in tuberculous constrictive pericarditis. Poor preoperative NYHA functional class and high preoperative CVP were shown to predict postoperative complication which was the predictor of prolonged ICU stay.

## Introduction

Tuberculosis is one of the globally significant infectious diseases with a high incidence and is the leading cause of infection related deaths, especially in developing countries [1]. In the low and middle income areas, tuberculosis is the most common cause of constrictive pericarditis with the reported incidence ranging from 23 to 91% [2, 3]. Surgical intervention has been recommended if there has been persistent clinical evidence of pericardial constriction after anti-tuberculous therapy [4]. Pericardiectomy is the standard surgical method for constrictive pericarditis [5]. In spite of its satisfactory effect in relieving pericardial constriction, pericardiectomy is accompanied by high risk of postoperative complication and in-hospital mortality [6–8].

In current clinical practice, the extent of pericardiectomy is varied according to surgeons’ experience and surgical difficulty. However, complete pericardiectomy has been reported to be superior to partial pericardiectomy in the improvement of hemodynamics and prognosis [9, 10], while the factors influencing the outcomes after pericardiectomy still remain unclear, especially in tuberculous constrictive pericarditis. This study aimed to identify the determinants of postoperative complication and prolonged intensive care unit (ICU) stay after pericardiectomy in tuberculous constrictive pericarditis.

## Methods

### Patients selection

There were one hundred and eight patients who were diagnosed as tuberculous constrictive pericarditis in our department from August 2012 to August 2019. We retrospectively reviewed their records and included the patients undergoing pericardiectomy in this study. The diagnosis of tuberculous pericarditis was based on bacteriological detection, nucleic acid detection and pathological examination. The diagnosis of constrictive pericarditis was determined mainly from clinical symptoms, imaging examinations and central venous pressure (CVP). Finally, a total of 88 patients were enrolled and their clinical characteristics were collected from the hospital electronic medical records system. The study protocol was approved by the Institutional Review Board of Hangzhou Red Cross Hospital.

### Perioperative process

Preoperative preparations included routine blood test, cardiopulmonary function assessment and imaging examination. The evaluation of pericardium mainly depended on cardiac ultrasound and contrast-enhanced computed tomography. Percutaneous internal jugular vein puncture and catheterization were performed preoperatively to measure the CVP.

All patients underwent general anesthesia and were placed in horizontal position. Median sternotomy was routinely performed in all cases without the use but with the preparation of cardiopulmonary bypass. The extent of complete pericardiectomy was defined as the resection of anterolateral pericardium between the two phrenic nerves including the pericardium lying on the ventricles, the basal pericardium over the diaphragmatic surface, the pericardium on the great arteries and the pericardium from superior vena cava-right atrium junction to inferior vena cava-right atrium junction [7, 11]. Other less extent was regarded as partial pericardiectomy. All patients were routinely admitted to ICU after surgery and transferred to the normal ward when the condition was steady. Postoperative complications were defined as the comorbidities that occurred after surgery but did not exist before.

### Statistical analysis

The enrolled patients were assigned to the postoperative complication group and the no postoperative complication group. The measurement data and numeration data of two groups were statistically analyzed with t test and χ2 test respectively. If the *P* value of any clinical characteristics was < 0.1, multivariate analysis was performed for those characteristics by the binary logistic regression to identify the factors predicting postoperative complication. Univariate and multivariate Cox regression analysis were performed to identify the independent risk factors of prolonged ICU stay. The length of ICU stay was compared between the two groups by the Kaplan-Meier curves and the log-rank test to further analyzed the association between the length of ICU stay and postoperative complication. All the above analysis was conducted by SPSS software (version 24.0, IBM SPSS Inc. United States). Statistical significance was set at *P* value < 0.05 (All *P* values presented were 2-sided).

## Results

### Postoperative complication and outcomes

We identified 88 patients with tuberculous constrictive pericarditis over the 7-year time period and all of them underwent complete pericardiectomy without the use of cardiopulmonary bypass. A total of 35 patients (39.8%) suffered postoperative complication (Table 1). Among these patients, the majority of cases (*N* = 23, 65.7%) had hypoalbuminemia. Low cardiac output occurred in 11 patients (31.4%), followed by pulmonary infection (*N* = 7, 20.0%). Arrhythmia occurred in 3 patients (8.6%) and wound infection in 2 (5.7%). Liver dysfunction was detected in 2 patients (5.7%) as well as hypokalemia (*N* = 2, 5.7%). Pulmonary embolism occurred in 1 patient (2.9%). It should be noted that 16 patients (45.7%) had two or more postoperative complications.

**Table 1:** Postoperative complication after pericardiectomy in study patients

Complication	*N* = 35
Hypoalbuminemia^a^	23 (65.7%)
Low cardiac output^b^	11 (31.4%)
Pneumonia	7 (20.0%)
Atrial fibrillation	3 (8.6%)
Wound infection	2 (5.7%)
Liver dysfunction^c^	2 (5.7%)
Hypokalemia	2 (5.7%)
Pulmonary embolism	1 (2.9%)

Values presented as N (percentage)

There were 16 patients suffering two or more postoperative complications

^a^The definition of hypoalbuminemia was the level of albumin lower than 30 g/L

^b^The definition of low cardiac output included cardiac index less than 2.0 L/min/m2 and systolic blood pressure less than 90 mmHg, in conjunction with signs of tissue hypoperfusion in the absence of hypovolemia

^c^The definition of liver dysfunction included alanine aminotransferase > 50 U/L, aspartate aminotransferase > 40 U/L or rising of bilirubin in the blood

The short term outcomes of postoperative complication were showed in Table 2. The patients with postoperative complication had longer postoperative ICU stay (*P* < 0.001) and postoperative hospital stay (*P* < 0.001) than those without postoperative complication. The duration of chest drainage was also prolonged in the postoperative complication group (*P* < 0.001), while postoperative NYHA functional class of patients with postoperative complication was similar to that of those without postoperative complication (*P* = 0.085). It was notable that there was no mortality within 30 days after surgery and no in-hospital death.

**Table 2:** Outcomes of study patients stratified by postoperative complication status

Variables	Postoperative complication		*P* value
	Yes (*N* = 35)	No (*N* = 53)	
Postoperative ICU stay, days	4.8 ± 2.4	2.1 ± 1.1	< 0.001
Duration of chest drainage, days	17.3 ± 8.3	9.2 ± 2.9	< 0.001
Postoperative hospital stay, days	24.0 ± 10.1	14.1 ± 3.4	< 0.001
Postoperative NYHA functional class			0.085
I	20 (57.1%)	42 (79.2%)	
II	14 (40.0%)	10 (18.9%)	
III	1 (2.9%)	1 (1.9%)	
Mortality within 30 days	0 (0.0%)	0 (0.0%)	/
In-hospital deaths	0 (0.0%)	0 (0.0%)	/

Values presented as mean ± standard deviation for continuous variables and N (percentage) for categorical variables

*ICU* intensive care unit, *NYHA* New York Heart Association

### Risk factors of postoperative complication

A total of 35 (39.8%) and 53 (60.2%) patients were assigned to the postoperative complication group and the no postoperative complication group, respectively. The association between postoperative complication and perioperative characteristics were shown in Table 3. Statistical differences were observed in symptom duration (*P* = 0.014), preoperative NYHA functional class (*P* = 0.019), pulse rate (*P* = 0.009), preoperative CVP (*P* = 0.041), pleural effusion (*P* = 0.035) and serum sodium (*P* = 0.038).

**Table 3:** The analysis of perioperative characteristics predicting postoperative complication

Variables	Postoperative complication		*P* value	Multivariate analysis		
	Yes (*N* = 35)	No (*N* = 53)		OR	95%CI	*P* value
Sex			0.310			
Male	29 (82.9%)	39 (73.6%)				
Female	6 (17.1%)	14 (26.4%)				
Age, years	56.6 ± 18.4	52.7 ± 15.2	0.288			
Symptom duration, months	2.1 ± 2.1	3.8 ± 4.1	0.014	1.160	0.909–1.480	0.233
Preoperative NYHA functional class			0.019	4.051	1.558–10.533	0.004
I	0 (0.0%)	7 (13.2%)				
II	9 (25.7%)	20 (37.7%)				
III	22 (62.9%)	25 (47.2%)				
IV	4 (11.4%)	1 (1.9%)				
Hypertension	5 (14.3%)	9 (17.0%)	0.735			
Diabetes	2 (5.7%)	3 (5.7%)	0.991			
Arrhythmia	8 (22.9%)	6 (11.3%)	0.148			
BMI, kg/m2	21.0 ± 2.9	21.8 ± 3.6	0.263			
SBP, mmHg	113.8 ± 14.0	116.7 ± 16.0	0.386			
DBP, mmHg	79.3 ± 12.0	79.1 ± 12.3	0.914			
Pulse rate (beats/min)	108.0 ± 15.5	99.0 ± 15.4	0.009	1.026	0.982–1.073	0.253
Preoperative CVP, cmH_2_O	27.9 ± 5.8	25.2 ± 5.3	0.041	1.151	1.013–1.309	0.031
Pleural effusion			0.035	6.057	0.675–54.367	0.108
Unilateral Bilateral	2 (5.7%) 33 (94.3%)	8 (15.1%) 39 (73.6%)				
Ascites	20 (57.1%)	27 (50.9%)	0.568			
Pericardial effusion	27 (77.1%)	41 (77.4%)	0.981			
Pericardial calcification	10 (28.6%)	12 (22.6%)	0.530			
Pericardial thickness, mm	11.2 ± 2.9	9.8 ± 3.4	0.055	1.126	0.902–1.406	0.293
LVEF, %	58.5 ± 6.9	58.9 ± 7.1	0.759			
Hepatomegaly	1 (2.9%)	1 (1.9%)	0.767			
Hemoglobin, g/dl	124.9 ± 15.0	121.1 ± 14.8	0.248			
CRP, mg/L	23.9 ± 20.1	23.6 ± 30.8	0.956			
ESR, mm/h	27.7 ± 18.3	27.8 ± 27.9	0.995			
Albumin, g/L	31.6 ± 4.1	33.3 ± 5.1	0.087	1.011	0.852–1.201	0.898
Serum sodium, mmol/L	136.7 ± 3.1	138.2 ± 3.5	0.038	1.098	0.887–1.360	0.390
Serum potassium, mmol/L	4.0 ± 0.5	3.9 ± 0.5	0.466			
Serum creatinine, μmol/L	84.4 ± 16.8	79.8 ± 12.4	0.144			
Operative duration, min	249.5 ± 45.6	233.3 ± 64.9	0.202			
Blood loss, ml	199.7 ± 163.9	166.0 ± 86.5	0.212			
Intraoperative fluid infusion, ml	1842.9 ± 446.9	1779.3 ± 526.6	0.558			
Postoperative CVP, cmH_2_O	15.0 ± 6.6	13.6 ± 5.2	0.315			

Values presented as N (percentage) for categorical variables and mean ± standard deviation for continuous variables

*OR* odds ratio, *CI* confidence interval, *NYHA* New York Heart Association, *BMI* body mass index, *SBP* systolic blood pressure, *DBP* diastolic blood pressure, *CVP* central venous pressure, *LVEF* left ventricular ejection fraction (measured on echocardiogram), *CRP* C-reactive protein, *ESR* erythrocyte sedimentation rate

Multivariate logistic analysis was further performed for the characteristics whose *P* values were < 0.1 to identify the risk factors of postoperative complication. As shown in Table 3, symptom duration, pulse rate, pleural effusion, pericardial thickness, albumin and serum sodium were not statistically associated with postoperative complication. The results revealed that preoperative NYHA functional class (*P* = 0.004, OR 4.051, 95%CI 1.558–10.533) and preoperative CVP (*P* = 0.031, OR 1.151, 95%CI 1.013–1.309) were independent risk factors of postoperative complication.

### Factors associated with length of ICU stay

**Fig. 1:**
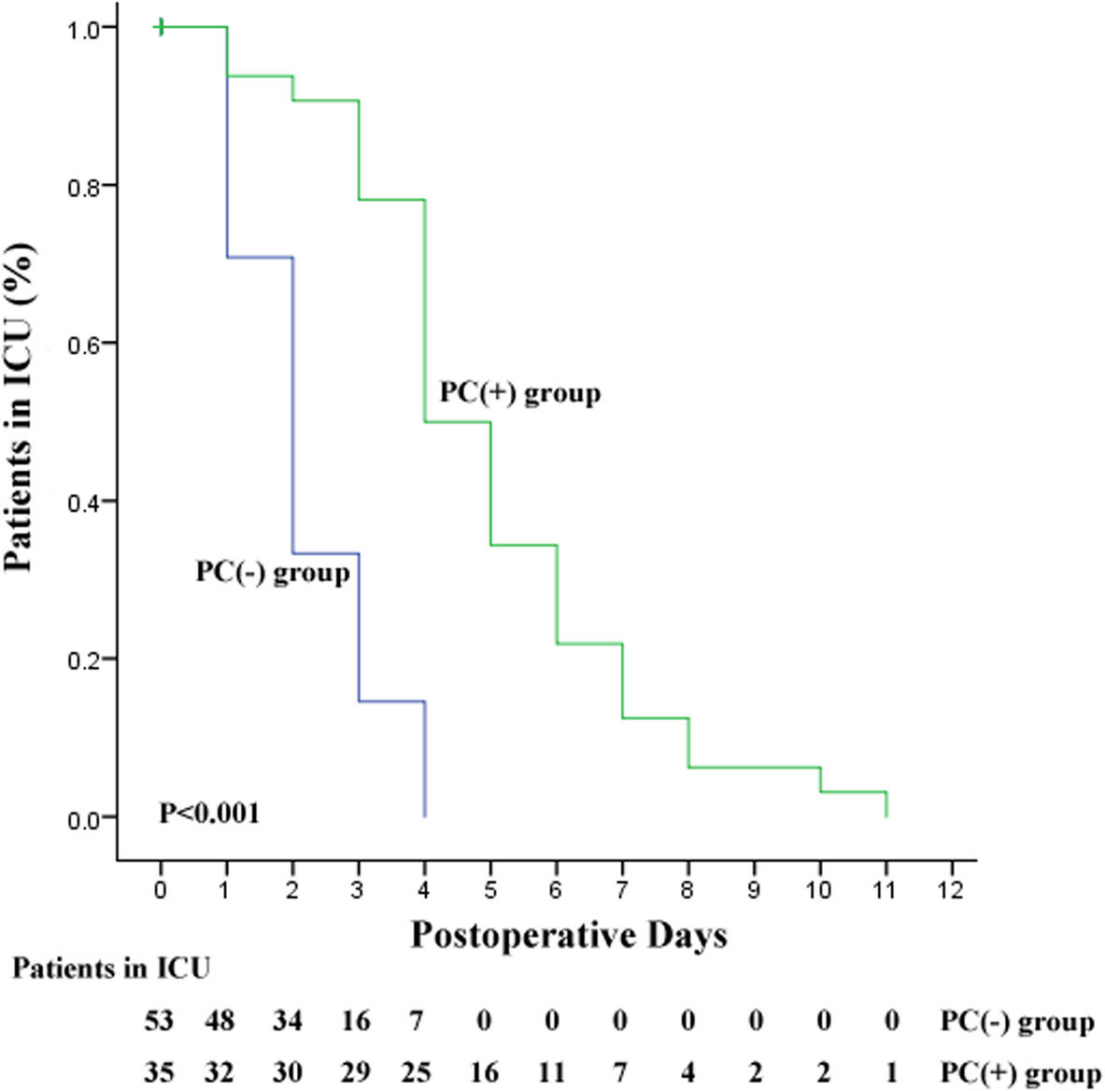
Kaplan–Meier curves comparing the length of ICU stay after surgery for the patients with and without postoperative complications. PC(+) group, the postoperative complication group; PC(−) group, the no postoperative complication group

The relationships between the perioperative factors and the length of ICU stay were analyzed with Cox regression model. Univariate Cox regression analysis showed that the length of ICU stay was significantly associated with age (*P* = 0.040), preoperative NYHA functional class (*P* = 0.042) and postoperative complications (*P* < 0.001). Additional multivariate Cox regression analysis proved that postoperative complication was the independent risk factor for prolonged ICU stay (*P* < 0.001, HR 4.132, 95%CI 2.217–7.692; Table 4). The ICU stay of two groups was compared by Kaplan–Meier curves (Fig. 1). The log-rank test showed that the patients with postoperative complication had longer ICU stay than those without postoperative complication (*P* < 0.001).

**Table 4:** Cox regression analysis of factors associated with the length of ICU stay

Variables	Univariate analysis			Multivariate analysis		
	*P* value	HR	95% CI	*P* value	HR	95% CI
Sex	0.639	1.139	0.662–1.957			
Age	0.040	1.014	1.001–1.027	0.559	1.004	0.990–1.019
Symptom duration	0.333	1.031	0.969–1.096			
Preoperative NYHA functional class	0.042	1.408	1.013–1.957	0.470	1.134	0.806–1.595
Hypertension	0.242	1.449	0.778–2.703			
Diabetes	0.481	1.387	0.558–3.448			
Arrhythmia	0.533	1.209	0.665–2.199			
Body weight	0.800	1.003	0.983–1.022			
SBP	0.424	1.007	0.990–1.024			
DBP	0.364	1.009	0.989–1.030			
Pulse rate	0.742	1.003	0.987–1.018			
Preoperative CVP	0.509	1.016	0.969–1.065			
Pleural effusion	0.255	1.285	0.834–1.980			
Ascites	0.164	1.380	0.877–2.171			
Pericardial effusion	0.953	1.016	0.599–1.724			
Pericardial calcification	0.897	1.034	0.620–1.727			
Pericardial thickness	0.807	1.009	0.938–1.085			
LVEF	0.534	1.012	0.975–1.051			
Hepatomegaly	0.482	2.043	0.280–14.927			
Hemoglobin	0.657	1.004	0.987–1.020			
CRP	0.481	1.005	0.992–1.017			
ESR	0.979	1.000	0.989–1.011			
Albumin	0.537	1.016	0.966–1.068			
Serum sodium	0.473	1.025	0.957–1.098			
Serum potassium	0.410	1.223	0.757–1.977			
Serum creatinine	0.542	1.005	0.989–1.021			
Operative duration	0.519	1.001	0.997–1.006			
Blood loss	0.145	1.001	1.000–1.003			
Intraoperative fluid infusion	0.642	1.000	1.000–1.001			
Postoperative CVP	0.480	1.016	0.972–1.063			
Postoperative complication	< 0.001	4.505	2.494–8.130	< 0.001	4.132	2.217–7.692

*HR* hazard ratio, *CI* confidence interval, *NYHA* New York Heart Association, *SBP* systolic blood pressure, *DBP* diastolic blood pressure, *CVP* central venous pressure, *LVEF* left ventricular ejection fraction (measured on echocardiogram), *CRP* C-reactive protein, *ESR* erythrocyte sedimentation rate

## Discussion

Constrictive pericarditis is an uncommon but potentially life-threatening disease. Because of the inflammatory disorder and fibrosis, pericardium becomes inelastic and then inhibits the cardiac filling. This process leads to the diastolic heart failure in the end with unfavorable clinical outcome [12]. Early surgical intervention was reported to play a positive role in reducing mortality rate [13, 14], but the diagnosis seems to be challenging in the early stage. The detection of constrictive pericarditis often relies on the typical clinical symptoms such as dyspnea and clinical signs such as jugular vein distention. Imaging examinations such as chest CT and cardiac MRI are important in the identification of pericardial effusion, calcification and thickening [12, 14, 15]. Meticulous echocardiographic examination is also valuable in the assessment of pericardial condition. Invasive haemodynamic catheterization and pressure measurement were of great significance in the diagnostic confirmation and the evaluation of the constrictive extent [16].

The definitive treatment for constrictive pericarditis is surgical pericardiectomy [17]. The surgical methods are classified as complete pericardiectomy and partial pericardiectomy according to the extent of pericardial resection. Complete pericardiectomy has been proven to be not only associated with lower perioperative mortality [9] but also confer significant long-term survival benefit and clinical functional improvement [10, 18]. Generally, pericardiectomy can be performed through either median sternotomy or left anterolateral thoracotomy, while median sternotomy provides adequate exposure of the right atrium, right ventricle and the vena cava, thus enabling extensive pericardial resection [19].

Despite the undoubtable effectiveness in treating constrictive pericarditis, pericardiectomy is associated with high risk of postoperative complication and mortality. A nationwide study in US revealed that the in-hospital complication and mortality rates after pericardiectomy were approximately 48 and 8%, respectively [8]. Also, Tokuda, Y. and his colleagues conducted a nationwide study on the outcome of pericardiectomy for constrictive pericarditis in Japan which showed the operative mortality was 10% and the major morbidity such as bleeding requiring reoperation was 15% [6]. In addition, Busch, C. et al. reviewed 97 consecutive patients undergoing surgery for constrictive pericarditis and reported that 1-year and 5-year survival rates were 66.5 and 51.6%, respectively [20]. Another retrospective study including 98 cases showed 1-year, 5-year, and 10-year survival rates were 82.5, 64.3, and 49.2%, respectively [21]. Although there are many researches about the surgical treatment for constrictive pericarditis in developed countries, the studies on tuberculous constrictive pericarditis have been limited in recent years due to the decreased incidence of tuberculosis worldwide. However, tuberculosis still remains the major etiology of constrictive pericarditis in developing countries [2, 3].

We have analyzed the short-term outcome of the patients with tuberculous constrictive pericarditis undergoing complete pericardiectomy over 7 years in our department. Although nearly 40% of patients in our study suffering postoperative complication, there was no mortality within 30 days after surgery and no in-hospital death. Hypoalbuminemia was the major postoperative complication possibly because of the negative nitrogen balance after surgery. The incidence of low cardiac output was also high enough to warrant attention, because it was proven to be the major contributor to in-hospital death in other studies [3, 22, 23]. In our study, postoperative complication was seemed to be associated with symptom duration, preoperative NYHA functional class, pulse rate, preoperative CVP, pleural effusion and serum sodium, while multivariate analysis eventually proved that poor preoperative NYHA functional class and high preoperative CVP were independent risk factors of postoperative complication, which might provide a valuable guidance for preoperative preparation and risk evaluation. We also found that postoperative complication significantly prolonged the postoperative ICU stay, duration of chest drainage and postoperative hospital stay. Additionally, postoperative complication was the independent risk factor for prolonged ICU stay. It should be emphasized that all patients undergoing complete pericardiectomy in our department were not routinely performed cardiopulmonary bypass, which was also proven to be safe in other studies [24, 25].

There are several limitations in this study. First, this is a single-center retrospective research that inevitably has the selection bias. Secondly, some important data such as duration of anti-tuberculosis medication, rate of prolonged intubation and duration of inotropic medication are unavailable due to the retrospective design. Finally, survival outcomes only include the mortality within 30 days after surgery and in-hospital deaths. Long-term outcome is required to be analyzed in the future.

## Conclusions

This study showed that complete pericardiectomy was associated with high risk of postoperative complication despite its benefits. Poor preoperative NYHA functional class and high preoperative CVP were shown to predict postoperative complication which was the predictor of prolonged ICU stay. This study has confirmed the need for early surgical intervention before the symptoms deteriorate to poor NYHA class or diastolic heart failure.

## Data Availability

The datasets used and/or analyzed during the current study are available from the corresponding author on reasonable request.

## Abbreviations

CVP: Central venous pressure
ICU: Intensive care unit
CT: Computed tomography
MRI: Magnetic resonance imaging
NYHA: New York Heart Association
BMI: Body mass index
OR: Odds ratio
CI: Confidence interval
SBP: Systolic blood pressure
DBP: Diastolic blood pressure
LVEF: Left ventricular ejection fraction
CRP: C-reactive protein
ESR: Erythrocyte sedimentation rate
HR: Hazard ratio
